# Chromosomal radiosensitivity as a marker of predisposition to common cancers?

**DOI:** 10.1054/bjoc.2000.1701

**Published:** 2001-04

**Authors:** K Baria, C Warren, S A Roberts, C M West, D Scott

**Affiliations:** CRC Cancer Genetics and Experimental Radiation Oncology^3^Groups and Biostatistics Unit; Paterson Institute for Cancer Research, Christie Hospital NHS Trust, Manchester, M20 9BX; Groups and Biostatistics Unit

**Keywords:** radiosensitivity, chromosome aberrations, inherited predisposition, colorectal cancer, lung cancer, cervical cancer

## Abstract

We previously found that 40% of breast cancer patients showed enhanced sensitivity to X-ray induced chromosome damage in G _2_ lymphocytes and suggested that this might indicate a low penetrance predisposition to breast cancer, for which there is good epidemiological evidence. We have now tested the hypothesis that elevated G _2_ radiosensitivity is a marker of such predisposition to other common cancers. We tested patients with colorectal cancer, for which there is also good epidemiological evidence of inherited risk in a substantial proportion of cases, and patients with cancers having a strong environmental aetiology (lung and cervix). We also repeated our study of breast cancer cases and tested patients with chronic diseases other than cancer. The results support our hypothesis, in that 30% (12/37) of colorectal cases showed enhanced sensitivity compared with 9% (6/66) of normal healthy controls (*P* = 0.01), whereas the proportions of sensitive cervix (11%, 3/27, *P* = 0.72) and lung cancer cases (23%, 8/35, *P* = 0.07) were not significantly above normals. We confirmed the enhanced sensitivity of 40% (12/31, *P* = 0.001) of breast cancer patients and found that patients with non-malignant disease had a normal response in the assay (12%, 4/34, *P* = 0.73). We suggest that enhanced G _2_ chromosomal radiosensitivity is a consequence of inherited defects in the ability of cells to process DNA damage from endogenous or exogenous sources, of a type that is mimicked by ionizing radiation, and that such defects predispose to breast and colorectal cancer. © 2001 Cancer Research Campaign http://www.bjcancer.com


*British Journal of Cancer* (2001) **84**(7), 892–896
© 2001 Cancer Research Campaign
doi: 10.1054/ bjoc.2000.1701, available online at http://www.idealibrary.com on IDEAL®

http://www.bjcancer.com

### Chromosomal radiosensitivity as a marker of predisposition to common cancers?

**K Baria[1], C Warren[1], SA Roberts[2], CM West[3] and D Scott[1]**

CRC Cancer Genetics[1] and Experimental Radiation Oncology[3] Groups, and Biostatistics Unit[2], Paterson Institute for Cancer Research, Christie Hospital NHS Trust, Manchester M20 9BX

**Summary** We previously found that 40% of breast cancer patients showed enhanced sensitivity to X-ray induced chromosome damage in $G_2$ lymphocytes and suggested that this might indicate a low penetrance predisposition to breast cancer, for which there is good epidemiological evidence. We have now tested the hypothesis that elevated $G_2$ radiosensitivity is a marker of such predisposition to other common cancers. We tested patients with colorectal cancer, for which there is also good epidemiological evidence of inherited risk in a substantial proportion of cases, and patients with cancers having a strong environmental aetiology (lung and cervix). We also repeated our study of breast cancer cases and tested patients with chronic diseases other than cancer. The results support our hypothesis, in that 30% (12/37) of colorectal cases showed enhanced sensitivity compared with 9% (6/66) of normal healthy controls ($P$ = 0.01), whereas the proportions of sensitive cervix (11%, 3/27, $P$ = 0.72) and lung cancer cases (23%, 8/35, $P$ = 0.07) were not significantly above normals. We confirmed the enhanced sensitivity of 40% (12/31, $P$ = 0.001) of breast cancer patients and found that patients with non-malignant disease had a normal response in the assay (12%, 4/34, $P$ = 0.73). We suggest that enhanced $G_2$ chromosomal radiosensitivity is a consequence of inherited defects in the ability of cells to process DNA damage from endogenous or exogenous sources, of a type that is mimicked by ionizing radiation, and that such defects predispose to breast and colorectal cancer. © 2001 Cancer Research Campaign http://www.bjcancer.com

**Keywords**: radiosensitivity; chromosome aberrations; inherited predisposition; colorectal cancer; lung cancer; cervical cancer

We have shown that approximately 40% of breast cancer patients have an enhanced sensitivity to the chromosome-damaging effects of ionizing radiation, compared with 5–10% of normal healthy controls, when their lymphocytes are irradiated in vitro in the $G_2$ phase of the cell cycle (Scott et al, 1994, 1999). This elevated radiosensitivity has now been confirmed in 3 independent studies (Parshad et al, 1996; Patel et al, 1997; Terzoudi et al, 2000). Since $G_2$ radiosensitivity is a feature of many inherited cancer-prone conditions such as ataxia-telangiectasia, Li-Fraumeni syndrome and hereditary retinoblastoma (reviewed in Scott et al, 1999), we proposed that $G_2$ sensitive breast cancer cases had an inherited predisposition to cancer, mediated through low penetrance genes, in contrast to the highly expressed genes *BRCA1*, *BRCA2* and *TP53*, which confer a strong family history and account for less than 5% of all cases (Goldgar et al, 1996; Ford et al, 1998). There is good epidemiological evidence for the existence of low penetrance predisposition in a substantial proportion of patients (Teare et al, 1994; Lichtenstein et al, 2000; Peto and Mack, 2000).

We have used several approaches to test the hypothesis that $G_2$ chromosomal radiosensitivity is a marker for low penetrance predisposition to breast and other common cancers. One approach was to investigate the $G_2$ response of blood relatives of breast cancer patients, from which we found good evidence of heritability of radiosensitivity attributable to the Mendelian segregation of one or two genes in each family (Roberts et al, 1999; Scott et al, 2000). In parallel with these family studies, and reported here, we tested patients with chronic diseases other than cancer, to determine whether or not the enhanced lymphocyte sensitivity of breast cancer cases could simply be a consequence of their illness. Also, we tested patients with colorectal cancer, for which there is good evidence of low penetrance predisposition (Cannon-Albright et al, 1988; Lichtenstein et al, 2000) in addition to the high cancer risk associated with rare mutations in the *APC* and mismatch repair genes (Farrington and Dunlop, 1996). As a further test of the specificity of $G_2$ sensitivity for inherited predisposition, we investigated the response of patients with cancers for which there is evidence of a strong environmental aetiology; namely cervical cancer, linked to infection with the human papilloma virus (Munoz and Bosch, 1992) and lung cancer, strongly associated with tobacco smoking (reviewed by Levi, 1999).

*Received 10 October 2000*
*Revised 12 December 2000*
*Accepted 19 December 2000*

*Correspondence to:* CM Watt. E-mail: cwest@picr.man.ac.uk

### MATERIALS AND METHODS

### Donors

We have tested normal healthy controls ($n$ = 66), patients with benign (non-cancerous) chronic diseases (34), a group of breast cancer patients (31) to compare with our previous cases, and patients with cervix (27) colorectal (37) or lung (35) cancer.

Further details of the participants are given in Table 1. The normal donors comprised staff from this Institute or members of the general public. The patients with benign disease had either diabetes mellitus or chronic lung disease that was not malignant (e.g. chronic obstructive airways disease, cryptogenic fibrosing alveolitis, bronchiectasis) Those with cancer were patients from the Christie Hospital NHS Trust and were tested before they received radio- or chemotherapy.

**Table 1** Characteristics of participants and culture success rate

| Group | Number of successful cases | Male/female | Mean age ± SD (range) | Total number of samples | Success rate (%) |
|---|---|---|---|---|---|
| Normals | 66[a] | 22/44 | 38 ± 11 (20–61) | 150 | 75 |
| Benign disease | 34 | 18/16 | 60 ± 15 (21–81) | 57 | 60 |
| Breast | 31 | 31 | 56 ± 9 (36–79) | 40 | 78 |
| Cervix | 27 | 27 | 59 ± 16 (33–83) | 34 | 80 |
| Colorectal | 37 | 22/15 | 63 ± 11 (32–86) | 57 | 65 |
| Lung | 35 | 24/11 | 69 ± 9 (50–85) | 92 | 38 |

[a]Several repeats on 25 donors giving a total of 112 successful samples (see text).

**Table 2** Aberration yields in the various groups compared with normals

| Group | Number | Mean spontaneous yield ± SD | Significance[a] (*P*) | % sensitive | Significance[b] (*P*) | Mean induced yield ± SD | Significance[c] (*P*) |
|---|---|---|---|---|---|---|---|
| Normals | 66 | 1.4 ± 1.4 | – | 9 | – | 85.8 ± 10.8 | – |
| Benign | 34 | 0.7 ± 1.5 | 0.001 | 12 | 0.73 | 85.8 ± 13.7 | 0.90 |
| Breast | 31 | 0.8 ± 1.0 | 0.028 | 39 | 0.001 | 96.5 ± 23.9 | 0.042 |
| Cervix | 27 | 1.4 ± 1.5 | 0.91 | 11 | 0.72 | 84.6 ± 13.9 | 0.82 |
| Colorectal | 37 | 1.3 ± 1.5 | 0.50 | 30 | 0.011 | 91.5 ± 18.4 | 0.26 |
| Lung | 35 | 1.3 ± 1.5 | 0.80 | 23 | 0.072 | 92.0 ± 14.6 | 0.044 |

[a]Comparison of spontaneous yields with normals. [b]Comparison of % sensitive with normals. [c]Comparison of induced yields with normals.

All of the blood samples were from local donors. This was an important feature of the $G_2$ testing because we have previously found poorer reproducibility in the assay from samples received from distant sources and transported by courier (Scott et al, 1999).

### The $G_2$ assay

Full details are given in Scott et al (1999). Briefly, whole blood cultures were set up in pre-warmed (37˚C) and pre-gassed (5% $CO_2$/95% air) medium. One hour later, lymphocytes were stimulated with phytohaemagglutinin (PHA) and cultured for 70 h, at which time the culture medium was replaced, without centrifugation, with fresh medium. Cells were irradiated (or mock irradiated) at 72 h with 0.5 Gy 300 kV X-rays, colcemid was added 30 min later and at 90 min after irradiation culture vessels were plunged into ice chippings. Subsequent centrifugation, hypotonic treatment and fixation was carried out at 4˚C. From 1 h before irradiation to the time of harvesting, cultures were kept at 37˚C.

Metaphase preparations were made with standard procedures and Giemsa stained. Slides were randomized and coded for analysis and 50 metaphases were scored from both irradiated and control samples. The low frequency of aberrations in control samples was substracted from that in irradiated samples to give the induced yield. The majority of aberrations were chromatid breaks and gaps that were greater than the width of the chromatid. Smaller achromatic lesions (gaps) were ignored (Sanford et al, 1989).

The proportion of blood cultures that yielded 50 analysable metaphases was less than in our previous studies (Scott et al, 1998, 1999; Roberts et al, 1999). For normal donors we had a 75% success rate (Table 1) compared with >95% previously. The success rate for patients with benign or malignant disease varied from 38% for lung cancers to 80% for cervix cancers (Table 1). There are several possible reasons, including the limited previous experience in cytogenetic procedures of the staff involved in laboratory aspects of this study. In addition, in contrast to our previous studies on breast cancer cases who were relatively healthy at the time of diagnosis and testing, many patients in this series were in more advanced stages of malignancy, often with co-morbid disease, particularly the lung and colorectal cases. Such morbidity is known to reduce the response of lymphocytes to PHA (Han and Takita, 1972; Catalona et al, 1973). Our interpretation of the results presented in this paper assumes that the degree of chromosomal radiosensitivity of an individual is not related to the likelihood of successful culturing of their lymphocytes.

### Statistical methods

Assay reproducibility was assessed using one-way analysis of variance to give values for inter-and intra-individual variability. Non-parametric Mann–Whitney U and Kruskall–Wallis tests were used to compare the patients with normals. Fisher's exact tests were used in comparisons of proportions of sensitive individuals. Correlations between parameters were assessed using Spearman's correlation coefficients. A significance level of 0.05 was used throughout.

### RESULTS

Numbers of successful assays, and ages and sexes of participants are given in Table 1.

### Normal donors

Results were obtained on 66 normals, 41 tested once, 25 tested on 2–6 occasions, giving a total of 112 successful tests.

The mean spontaneous aberration yield was 1.4 ± 1.4 aberrations/100 cells (Table 2), similar to our previous estimate of 1.8 ± 1.9 for 105 normals (Scott et al, 1999).

The mean induced frequency of aberrations was 85.8 ± 10.8 per 100 cells (range 64–118, Figure 1), slightly lower than our previous value of 97 ± 15 (range 75–163; Scott et al, 1999). This is probably because different microscopists were involved in these studies and reflects small differences in scoring criteria. The repeat assays on 25 donors allowed an estimate of intra-individual

*© 2001 Cancer Research Campaign* 

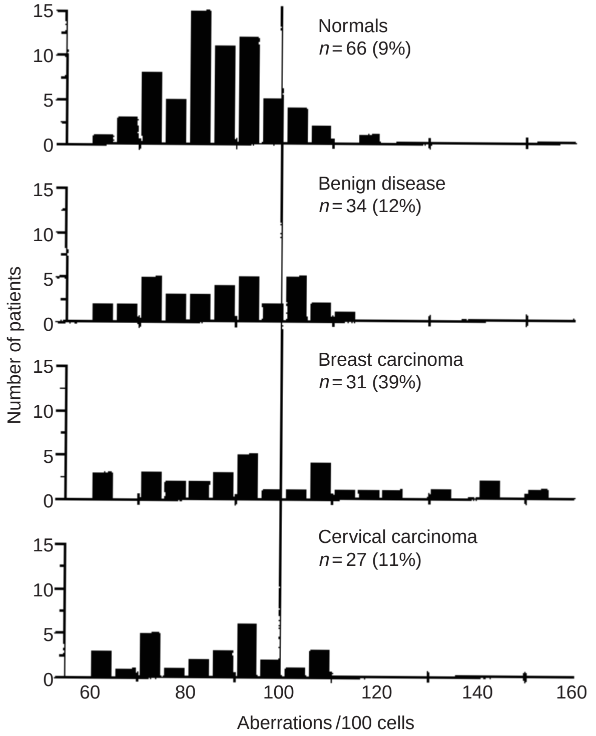

**Figure 1** Yields of radiation-induced aberrations in normal donors and in patients with non-malignant disease, breast carcinoma and cervical carcinoma. Repeat tests were performed on 25 normals (2–6 repeats), giving a total of 112 samples. The vertical solid line indicates the cut-off point between a normal and a sensitive response. The percentage of sensitive individuals is given in brackets

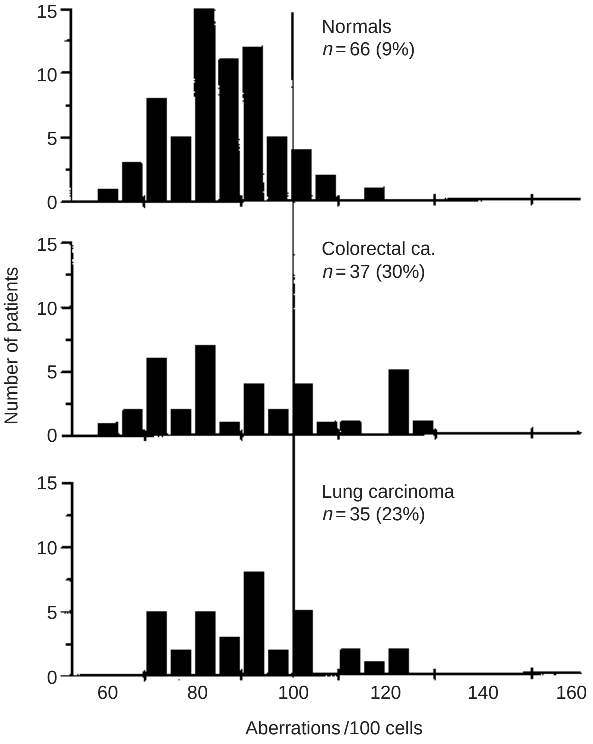

**Figure 2** As for Figure 1 but for normals, colorectal carcinoma and lung carcinoma patients variance (assay reproducibility) which gave a coefficient of variation (CV) of 10.3% compared with a CV of 15.1% for inter-individual differences between donors. This indicated a significant difference between donors ($P$ = 0.004) as we have previously demonstrated (Scott et al, 1999). There was no significant influence of age (r = 0.11, $P$ = 0.39) or sex ($P$ = 0.96) on induced yields, as reported earlier (Scott et al, 1999).

### Patients

None of the patient groups had spontaneous aberration frequencies that were above the normals. In fact, the yields in the breast cancer cases and benign disease group were less than in normals (Table 2). We previously found no difference between 135 breast cancer patients and 105 normals (Scott et al, 1999). The lower yields in the benign group in this study is probably a chance finding, reflecting the relatively small number of cases tested.

Radiation-induced aberration frequencies are given in Table 2 and in Figures 1–2. For none of the patient groups was there any significant influence of age or sex on induced aberration frequencies.

Only for breast, colorectal and lung cancer patients were mean induced aberration yields higher than in normals, but only for breast cases was this increase statistically significant (Table 2). A better method of comparing patients with normals is to express the results in terms of the proportion of sensitive/non-sensitive cases using the 90th percentile of normals as the cutoff value. This follows from our previous studies of the heritability of $G_2$ sensitivity in which we found that the distribution of $G_2$ values was multimodal and that the use of the 90th percentile cutoff distinguished well between normal individuals and those carrying putative radiosensitizing genes (Roberts et al, 1999). In the present study this cutoff value was at 100 aberrations/100 cells (Figure 1). This value actually gave a sensitive proportion of 9% (6/66), rather than exactly 10%, because the yields for several normals fell exactly at the cut-off point. The proportion of sensitive cases was significantly higher for breast (39%) and colorectal (30%) cancer patients (Table 2, Figures 1 and 2). For lung cancer patients, 23% were sensitive (Figure 2), but this increase did not reach statistical significance ($P$ = 0.07). Patients with benign disease or cervix cancer had sensitive proportions that were very similar to normals (Fig. 1).

### DISCUSSION

Our results are consistent with the hypothesis that elevated $G_2$ lymphocyte chromosomal radiosensitivity is a marker of low penetrance predisposition to common cancers.

Our observation that patients with diabetes or chronic non-malignant lung disease show a normal response makes it less likely that enhanced sensitivity in the assay is simply a consequence of morbidity, as does our demonstration that the mean sensitivity of healthy blood relatives of breast cancer patients is greater than that of normals (Roberts et al, 1999). However, patients with other benign diseases should be tested to further explore this possibility.

We have confirmed our earlier observations (Scott et al, 1994, 1999) that approximately 40% of breast cancer patients are

 *© 2001 Cancer Research Campaign*

$G_2$-sensitive. The elevated sensitivity of breast cancer patients is now well established, having been demonstrated in 3 independent studies (see earlier). Further epidemiological evidence that a high proportion of breast cancers arise in genetically predisposed individuals has been provided by studies of breast cancer in twins (Peto and Mack, 2000).

Our hypothesis predicts that the enhanced $G_2$ sensitivity that we observed in 30% of colorectal cancer cases is indicative of low-penetrance predisposition. Family studies by Cannon-Albright et al (1988) strongly support the existence of such genes and led to their suggestion that inherited susceptibility is involved in a high proportion of colorectal cancers. From analyses of cancers in twins in Scandinavia, Lichtenstein et al (2000) conclude that 35% of the risk of colorectal cancer can be explained by heritable factors.

The lack of a statistically significant increase in the proportion of $G_2$-sensitive lung cancer cases compared with normals is consistent with the knowledge that cancer at this site has a predominantly environmental aetiology and is largely a consequence of tobacco useage. However, there are also indications of genetic predisposition to lung cancer (reviewed by Sellers, 1996; Lichtenstein et al, 2000) with some evidence that smoking-related cases are associated with polymorphisms in *CYP2* genes involved in the metabolic activation of procarcinogens in cigarette smoke (Uematsu et al, 1991). If differences in carcinogen metabolism underlie susceptibility to lung cancer, these differences would not be detected by the $G_2$ assay. The increase in proportion of sensitive lung cancer cases, although non-significant, may indicate a reduced DNA repair capacity in some patients. Rudiger et al (1989) found that levels of the DNA repair enzyme 06-methylguanine-DNA-methyltransferase were, on average, lower in cells of lung cancer patients than in healthy controls. However, this enzyme is involved in the repair of damage from alkylating agents and there is no reason to assume that it would influence response to ionizing radiation.

Patients with colorectal or lung cancer show enhanced sensitivity to the chromosome-damaging effects of bleomycin in $G_2$ lymphocytes (Hsu et al, 1989). However, breast cancer patients were similar to normals, so the response of $G_2$ cells to bleomycin cannot be regarded as a surrogate for $G_2$ radiation sensitivity.

Although there is evidence that risk of cervical cancer is associated with specific histocompatibility antigens which confer reduced immune surveillance of human papillomavirus (Stern, 1996) such immune deficiency would not be detected with our radiosensitivity assay and this is reflected in our results. The twin studies of Lichtenstein et al (2000) did not show any significant inherited component in the risk of cervical cancer.

Evidence that a particular type of cancer is caused by specific environmental exposure does not preclude an inherited component of risk which *would* be detected by the $G_2$ assay. For example, we have recently shown in head and neck cancers, for which there is strong evidence of causation by tobacco and alcohol consumption, there is increased $G_2$ sensitivity in early onset cases (Papworth et al, 2001).

Recently, Terzoudi et al (2000) reported that the average $G_2$ radiosensitivity of 185 patients with various cancers was significantly greater than that of 25 normals. The patients included 14 with lung cancer and 20 with cervix cancer, both groups having elevated mean yields of aberrations compared with the normals. However, the statistical significance of these increased yields was not given. Importantly, the range of yields for the normals, and for the lung and cervix cancer patients, was 2–3 times greater than we have observed so the numbers of individuals tested by Terzoudi et al may be insufficient to establish whether or not there is a clear enhancement in these patients.

Studies of heritability of $G_2$ sensitivity of the type that we have undertaken for breast cancer patients would help to clarify the predictive value of the assay for cancer predisposition at other sites. Where heritability can be demonstrated, this could lead to the identification of the predisposing genes by genetic linkage analysis (see Roberts et al, 1999).

### ACKNOWLEDGEMENTS

We wish to thank all the blood donors, Mrs Ann Spreadborough for tuition in the assay and the following clinicians for co-operation in obtaining blood samples from their patients; Drs P Barber, P Burt, S Davidson, E Levine, B Magee, J Manns and M Saunders. Funding was provided by the Cancer Research Campaign and the UK Co-Ordinating Committee on Cancer Research.

### REFERENCES

Cannon-Albright LA, Skolnick MH, Bishop T, Lee RG and Burt RW (1988) Common inheritance of susceptibility to colonic adenomatous polyps and associated colorectal cancers. *New Eng Med J* **319**: 533–537

Catalona WL, Sample WF and Chretien PB (1973) Lymphocyte reactivity in cancer patients: correlation with tumour histology and clinical stage. *Cancer* **31**: 65–71

Farrington SM and Dunlop MG (1996) In: *Genetic Predisposition to Cancer*, Eeles RA, Ponder BAJ, Easton DF and Horwich A (eds), pp 306–319. Chapman & Hall: London

Ford D, Easton DF, Stratton M, Narod S, Goldgar D, Devilee P and Bishop DT et al (1998) Genetic heterogeneity and penetrance analysis of the BRCA1 and BRCA2 genes in breast cancer families. *Am J Human Genet* **62**: 676–689

Goldgar DE, Stratton MR and Eeles RA (1996) Familial breast cancer. In: *Genetic Predisposition to Cancer*, Eeles RA, Ponder BAJ, Easton DF and Horwich A (eds), pp 227–238. Chapman & Hall: London

Han T and Takita H (1972) Immunologic impairment in bronchogenic carcinoma: A study of response to phytohaemagglutinin. *Cancer* **30**: 616–619

Hsu TC, Johnston DA, Cherry LM, Ramkissoon D, Schantz SP, Jessup JM, Winn RJ, Shirley L and Furlong C (1989) Sensitivity to genotoxic effects of bleomycin in humans: possible relationship to environmental carcinogenesis. *Int J Cancer* **43**: 403–409

Levi F (1999) Cancer prevention: Epidemiology and perspectives. *Eur J Cancer* **35**: 1046–1058

Lichtenstein P, Holm NV, Verkasalo PK, Iliado A, Kaprio J, Koskenvuo M, Pukkala E, Skytthe A and Hemminki K (2000) Environmental and heritable factors in the causation of cancer. Analyses if cohorts of twins from Sweden, Denmark and Finland. *New Eng Med J* **343**: 78–85

Munoz N and Bosch F (1992) HPV and cervical neoplasia: Review of case-control and cohort studies. In: *The epidemiology of cervical cancer and human papillomavirus*, Munoz N, Bosch FX, Shah KV and Meheus A (eds), pp 251–261. International Agency for Research on Cancer: Lyon

Papworth R, Slevin N, Roberts SA and Scott D (2001) Sensitivity to radiation-induced chromosome damage may be a marker of genetic predisposition in young head and neck cancer patients. *Br J Cancer* (in press).

Parshad R, Price FM, Bohr VA, Cowans KH, Zujewski JA and Sanford KK (1996) Deficient DNA repair capacity, a predisposing factor in breast cancer. *Br J Cancer* **74**: 1–5

Patel RK, Trevedi AH, Arora DC, Bhatavdekar JM and Patel DD (1997) DNA repair proficiency in breast cancer patients and their first degree relatives. *Int J Cancer* **73**: 20–24

Peto J and Mack TM (2000) High constant incidence in twins and other relatives of women with breast cancer. *Nature Genetics* **26**: 411–414

Roberts SA, Spreadborough AR, Bulman B, Barber JBP, Evans DGR and Scott D (1999) Heritability of cellular radiosensitivity: a marker of low penetrance predisposition genes in breast cancer? *Amer J Human Genet* **65**: 784–794

Rudiger HW, Schwartz U, Serrand E, Matthias S, Krause T, Nowak D, Doerjer G and Lehnert G (1989) Reduced O6-methylguanine repair in fibroblast cultures from patients with lung cancer. *Cancer Res* **49**: 5623–5626

© 2001 Cancer Research Campaign

Sanford KK, Parshad R, Gantt RE, Tarone RE, Jones GM and Price FM (1989) Factors affecting and significance of G2 chromatin radiosensitivity and predisposition to cancer. *Int J Radiat Biol* **55**: 963–968

Scott D, Spreadborough A, Levine E and Roberts SA (1994) Genetic predisposition to breast cancer. *Lancet* **344**: 1444

Scott D, Barber JB, Levine EL, Burrill W and Roberts SA (1998) Radiation-induced micronucleus induction in lymphocytes identifies a high frequency of radiosensitive cases among breast cancer cases: a test for predisposition? *Br J Cancer* **77**: 614–620

Scott D, Barber JBP, Spreadborough AR, Burrill W and Roberts SA (1999) Increased chromosomal radiosensitivity in breast cancer patients: a comparison of two assays. *Int J Radiat Biol* **75**: 1–10

Scott D, Roberts SA, Spreadborough, Bulman B, Barber JBP and Evans DGR (2000) Chromosomal radiosensitivity and cancer predisposition. In: *Radiation Research, Volume 2. Congress Proceedings*, Moriarty M, Mothershill C, Seymour C, Edington M, Ward JF and Fry RJM (eds). 11th International Congress of Radiation Research, pp 470–471. Allen Press: Lawrence, USA

Sellers TA (1996) Familial predisposition to lung cancer. In: *Genetic Predisposition to Cancer*, Eeles RA, Ponder BAJ, Easton DF and Horwich A (eds), pp 344–353. Chapman & Hall: London

Stern PL (1996) Immunity to human papillomavirus-associated cervical cancer. In: *Advances in Cancer Research* **6**, Klein G and Vande Woulde GF (eds), pp 175–211. Academic Press: New York

Teare MD, Wallace SA, Haris M, Howell A and Birch JM (1994) Cancer experience in the relatives of an unselected series of breast cancer patients. *Br J Cancer* **10**: 102–111

Terzoudi GI, Jung T, Hain J, Vrouvas J, Margaritis K, Donta-Bakoyiannis C, Makropoulos V, Angelakis PH and Pantelias GE (2000) Increased chromosomal radiosensitivity in cancer patients: the role of cdk1/cyclin-B activity level in the mechanisms involved. *Int J Radiat Biol* **76**: 607–615

Uematsu F, Kikuchi H, Motomiya M, Abe T, Sagami I, Ohmachi T, Wakui A, Kanamaru R and Watanabe M (1991) Association between restriction fragment polymorphism of the human cytochrome p450IIE1 gene and susceptibility to lung cancer. *Jpn J Cancer Res* **82**: 254–256

 *© 2001 Cancer Research Campaign*
